# Polymorphisms in the IL-6 and IL-6R receptor genes as new diagnostic biomarkers of acute appendicitis: a study on two candidate genes in pediatric patients with acute appendicitis

**DOI:** 10.1186/s13052-015-0206-7

**Published:** 2015-12-29

**Authors:** Sevgi Büyükbeşe Sarsu, Şenay Görücü Yılmaz, Ali Bayram, Affan Denk, Kürşat Kargun, Mehmet Ali Sungur

**Affiliations:** Department of Pediatric Surgery, Gaziantep Children Hospital, Gaziantep, 27560 Turkey; Department of Nutrition and Dietetics, Faculty of Health Sciences, Gaziantep University, Gaziantep, 27310 Turkey; Department of Medical Biology and Genetics, Elazığ High School of Health Sciences, Fırat University, Elazığ, 23100 Turkey; Department of Infectious Diseases and Clinical Microbiology, Faculty of Medicine, Fırat University, Elazığ, 23100 Turkey; Department of Biostatistics and Medical Informatics, Faculty of Medicine, Düzce University, Düzce, 81620 Turkey

**Keywords:** Appendicitis, Interleukin-6, Interleukin-6 receptor, Polymorphism, Disease progression

## Abstract

**Background:**

Acute appendicitis (AA) (OMIM: 107700) is an inflammatory disease which is characterized by appendiceal inflammation. Genetic and environmental factors contribute to the development of AA. Especially, multiple genetic factors appear to be promising in the explanation of etiopathogenesis of AA. IL-6 (Interleukin-6) is an inflammatory cytokine and IL-6 receptor (IL-6R) plays an important role in the immune response. IL-6 (-572G/C rs1800796) and IL-6R (1:G.154448302 T > C rs7529229) gene polymorphisms may have an impact on cytokine production, immune response and these gene polymorphisms may be used as inflammatory markers in the diagnosis of appendicitis.

**Method:**

A total of 75 children with appendicitis, and 75 healthy children were included in the study. DNA extracts were obtained from peripheral lymphocytes. Single-nucleotide polymorphisms (SNPs) were analysed using an automated **SYBR**® Green RT-**PCR** system in pediatric patients with appendicitis (*n* = 75) and healthy controls (*n* = 75).

**Results:**

The allele and genotype frequencies for IL-6 rs1800796 and IL-6R rs7529229 polymorphisms were not different between the study groups (*p* > 0.05). Any statistically significant differences as for age, sex and other laboratory factors were not detected between the patients with appendicitis for genotype-allele frequencies (*p* > 0.05). Still in analyses performed to determine correlations among age, and gender of the patients, routine laboratory parameters and allele-genotype frequencies, a statistically significant intergroup difference was not detected. Genotype and allele frequencies were consistent with Hardy-Weinberg equilibrium (HWE) in all groups.

**Discussion:**

This is the first study to investigate the effects of functional two polymorphisms on IL-6 and IL-6R genes in a pediatric patient group with AA risk. With this study we investigated the contribution of IL-6 (-572G/C rs1800796) and IL-6R (1:G.154448302 T > C rs7529229) polymorphisms on pathogenesis, and severity of AA in pediatric patients with AA: These results will guide further genetic researches to be performed on the role of IL-6 and IL-6R in AA.

**Conclusions:**

Given the putative biological importance of this SNPs, these emerging data can provide a new foundation to stimulate future debate and genetic investigations of AA, focusing on new molecular mechanisms such as other IL gene polymorphisms, particularly in accessible peripheral tissues for novel molecular diagnostics for appendicitis.

## Background

Acute Appendicitis (AA) is a condition characterized by inflammation of the appendix accompanied by systemic inflammatory response [1]. AA is observed in about 7 % of the population [2, 3]. Appendectomy was firstly described by Claudius Amyand 270 years ago, and from then many universally accepted effective treatment methods have been introduced. Appendectomy is the most common type of emergency surgery with an incidence rate of 1-2 % [4, 5]. This disease is mostly identified in adolescents. Its etiopathogenesis is not very explicit. The most widely accepted view concerning the development of the disease is luminal occlusion with fecaloids, however fecaloids were not observed in nearly 30–40 % of the samples harvested [6]. When human appendix is compared with the other areas of colon, its microbial content is much higher. Development of edema in the appendiceal tissue and inflammatory changes result in rapid distension of the appendix with resultant increase in the intraluminal pressure. Lymphatic and venous drainage cease with the increased venous pressure which leads to the development of mucosal ischemia [7]. Researchers conducted so far have demonstrated the impact of age, gender, segregation, socio-economic status, nutritional habits and genetic factors on the development of acute appendicitis [8, 9]. However, genetic predisposition is still very poorly understood [10]. Especially, in pediatric population establishment of diagnosis is quite difficult. Although many imaging techniques aid in its diagnosis, a significant decrease has not been observed in the incidence rates of negative appendectomy and perforated appendicitis. Appendiceal inflammation manifests itself from acute appendicitis to necrosis and perforation. Also, some appendectomy specimens are evaluated histologically zaps normal appendiceal tissue samples. Immune response and inflammatory factors are important in the inflammatory appendix [11]. These factors demonstrate the importance of cytokines in determining the course of appendicitis. Concomitant evaluation of clinical and laboratory findings has a higher diagnostic value in cases with suspect diagnosis. Interleukin-6 (IL6) is a pro-inflammatory cytokine and it is an early indicator of tissue injury and systemic inflammatory response. The diagnostic value of IL6 which triggers acute phase response as release of neutrophils and CRP (C-reactive protein) is more important in diseases characterized by excessive production of acute phase proteins. Polymorphisms in the regulatory regions of cytokine genes clearly alter functions, and amount of cytokines produced [12]. Furthermore, various studies have demonstrated that these two polymorphisms are associated with many disease states such as abdominal aortic aneurysm, symptomatic distal interphalangeal osteoarthritis and obstructive sleep apnea [13–15]. Meta-analyses have also demonstrated the correlations between these polymorphisms and chronic inflammatory diseases [16]. IL6 rs1800796 promoter polymorphism is responsible for the transcription of these genes, and secretion of IL-6 cytokine. IL-6 is mainly produced by T cells, macrophages and adipocytes. IL-6 is needed for the initiation of IL6 signal transduction and the cytokine responses [17]. In this case, IL-6R involves in the receivable of this signal. IL-6R rs7529229 polymorphism may impair delivery of necessary signals for the cytokine response. Appendicitis is an inflammatory bowel disease and the severity of local inflammation influenced by polymorphisms in genes conferring congenital immunity. In this study, we examined the role of rs1800796 and rs7529229 functional polymorphisms in pediatric patients who underwent appendectomy with diagnosis of appendicitis. We think that these function polymorphisms can contribute to the severity of local inflammation occurring in pediatric patients with appendicitis.

## Methods

### Study population and epidemiologic data

The study population consisted of 75 (43 boys and 32 girls) appendectomized patients and 75 (32 girls and 43 boys) healthy aged 6-16 years, attending the Pediatric Surgery Department of Children’s Hospital of Gaziantep in Turkey within the time interval between June 2014 and January 2015. The patients with AA were diagnosed according to routine biochemical test (CRP, ESR, WBC, etc.) results, histopathological examination criteria and ultrasonography. The definitive diagnosis was established by histopathological examination. Control subjects had no AA history. We investigated the association between allele, and genotype frequencies and CRP values. The study protocol was approved by the institutional ethics committee of the Medical Faculty of Gaziantep University in Turkey. All volunteers signed an informed consent form before they entered into this research study. A 2 ml blood sample from each subject was drawn into a coded heparinized tube for genetic analyses.

#### Selection of candidate genes and functional SNPs

Effect of SNPs may be different in healthy and abnormal tissues and cells. This diversity may result in various disorders. The SNPs have been studied in other common complex diseases such as obesity, metabolic disorder but not in another complex disease as AA. With the intention to close this information gap, and also demonstrate the effect of SNPs in AA, we opted to investigate these genes, and overlying functional SNPs Ancestral allele for IL-6 rs1800796 (-572 G/C) is G, and the ratio between minor allele frequency and population size is 0.3139/1572 (MAF Source: 1000 Genomes). Frequencies of GG, GC and CC genotypes are different in healthy populations in Asia, Europe and Africa. Frequencies of GG, GC and CC genotypes are 0.914 (GG), 0.086 (GC), 0 (CC), respectively, while respective frequencies of G and C alleles are 0.957 and 0.043 in Europe population (HapMap-CEU. 116 chromosome). These results are different in Asian population (GG = 0.044, GC = 0.378, and CC = 0.578, respectively). Our study results demonstrate similarities to those conducted in European and African populations.

Ancestral allele for IL6-R rs7529229 T/C) is T and the ratio between minor allele frequency and population size is 0.4539/2273 (MAF Source: 1000 Genomes). TT, TC and CC genotype frequencies are similar in healthy populations in Asia, Europe and Africa and our study group.

### DNA extraction

Genomic DNA was extracted from peripheral blood with the QIAampDNA Mini Kit and spin-column method (QIAGENE, Germany) according to the manufacturer’s protocol. Samples were stored at −20 °C until analysis.

### Detection of polymorphisms

Genotyping of IL-6 rs1800796 and IL-6R rs7529229 SNPs were carried out by the Real-Time PCR system (Rotor-Gene Q, QIAGENE) with probe technology of SYBR Green (SYBR Green PCR Kit-204076). DNA was extracted according to manufacturer’s protocol (QIAampDNA Mini Kit - 51306) and amplified in a 20 μl final volume of SYBR Green PCR buffer containing 0.02 μmol/ μl of common primer for IL-6 (forward, 5′-GGA GTC ACA CAC TCC ACC TG-3′; reverse, 5′-TGT GTT CTG GCT CTC CCT GT-3′), IL-6R (forward, 5′-TAG AAA TGT GGT CGT GGT GAG-3′; reverse, 5′-CGC TGC TCC ACT CCT TAC-3′) and 200 ng genomic DNA. The protocol used for Real-Time PCR consisted of initial denaturation: 95 °C for 5 min; amplification step: 40 cycle of 95 °C for 5 s, 60 °C for 10 s, then 1 cycle of 65 °C > 95 °C for melting.

### Statistical analyses

Data were analysed using statistical package SPSS v22.0 for Windows operating system. The differences between groups were analysed by the Independent Samples -*t* test. The associations of genotypes and alleles were analysed using *chi*-square or likelihood ratio tests. Hardy-Weinberg equilibrium was checked for the patient and the control groups. Two-tailed p differences less than 0.05 were regarded as significant. The required minimum sample size was calculated to be approximately 50 individuals in each group in order to detect the difference between the appendicitis and the control groups under the conditions of 5 % Type I error and 80 % power (Type II error 0.20). Power analysis was performed with MedCalc software package v.11.3.5.

## Results

SNP frequencies on the IL-6 and IL-6R genes in patients and controls, and the different characteristics of patients with appendicitis are shown in Tables 1 and 2. The mean ages of the AA and control groups were 11.78 ± 2.25 and 10.97 ± 2.75 years, respectively. Distributions of these observed genotypes were not significantly different from the expected distributions according to the Hardy-Weinberg equilibrium (HWE). There were no differences in allele frequencies in the variants of IL-6 and IL-6R genes between patients and the controls (*p* > 0.05). Futhermore, no statistically significant differences between patients with appendicitis were not observed as for age, sex and other laboratory factors (*p* > 0.05). Additionally, no correlation was found between SNPs, genotype frequencies and CRP levels (data not shown).

**Table 1:** Genotype Distributions of SNPs in the Patient and Control Groups

Gene	SNP	Variant (M > m)	Genotype	Patient *N* (%)	Control *N* (%)	*p*-value
IL-6	rs1800796	G > C	GG	67 (89.3)	72 (96.0)	0.14
			GC	6 (8.0)	3 (4.0)	
			CC	2 (2.7)	0 (0.0)	
IL-6R	rs7529229	T > C	TT	69 (92.0)	73 (97.3)	0.24
			TC	5 (6.7)	2 (2.7)	
			CC	1 (1.3)	0 (0.0)	

*M* major allele, *m* minor allele, *N* number of individulas

**Table 2:** Significant Associations of SNPs in the Patient and the Control Groups

SNP	Genotype	OR (95 % CI)	*p*-value
rs1800796	GC	2.15 (0.52-8.94)	0,29
	CC	---	---
	GC + CC	2.87 (0.73-11.25)	0.13
	C	3.50 (0.94-12.98)	0,06
rs7529229	TC	2.66 (0.50-14.08)	0.25
	CC	---	---
	TC + CC	3.17 (0.62-16.26)	0.17
	C	3.62 (0.74-17.73)	0.11

*CI* confidence interval, *OR* odds ratio

## Discussion

AA is a disease characterized by acute suppurative infection. Early diagnosis and treatment are important. AA may cause systemic inflammation and severe complications such as perforation or sepsis over time. Genetic and environmental factors are responsible in AA etiology [18]. Nowadays, routine inflammatory markers together with ultrasonographic, and/or computed tomographic methods are used in the preoperative diagnosis of AA. However, the rates of negative appendectomy and perforated appendicitis still remain fairly high, around 15 % [19]. These ratios directly signify requirement of an exact marker in the diagnosis of AA [20].

IL-6 is one of the key cytokines of acute phase of inflammatory response [21] and plays an important role in immune cell maturation by induction of immunoglobulin production of B and differentiation of T cells. It is also essential for antibody production by B cells, induces IL-2 production and consequently T cell differentiation, activates endothelial cells and encourages chemokine production and adhesion molecule expression [22, 23]. Ozguner and colleagues determined that IL-6 levels may be useful in the prediction of the patients with appendicitis [24]. Similarly, Anielski and colleagues also suggested that IL-6 may be a useful marker in reducing the number of false-positive diagnoses of AA [25]. IL-6 572G/C polymorphism has been previously studied in some inflammatory conditions with conflicting results. The -572G/C polymorphism was determined to be significantly associated with chronic obstructive pulmonary disease susceptibility under a dominant model of inheritance [26]. The 572C allele showed an increased promoter activity in response to IL-1b and TNF-α in transfected cells which was associated with higher levels of C-reactive protein and systemic IL-6 activity [27]. Similarly serum concentrations of C-reactive protein in subjects homozygous for the 572G allele were significantly higher in smokers, suggesting that individuals with IL-6 572C > G have a greater susceptibility to the inflammatory effects of smoking in the long term [28]. Zhang and colleagues reported that there was not a significant difference in the distribution of the IL-6 572G/C polymorphisms between lung cancer patients and controls, although some other polymorphisms such as IL-6-1363 T/G, IL-10 -819 T/C and IL-10 -592A/C polymorphisms were more common in the patient group [29]. Similarly Zavaleta-Muñiz and colleagues could not detect correlation between 174G/C and -572G/C IL6 promoter gene polymorphisms and rheumatoid arthritis which is a condition with chronic inflammation [30]. IL-6R gene polymorphisms have been also reported to be associated with systemic immune response [31]. Wypasek and colleagues reported that IL-6R single nucleotide polymorphisms were associated with mean concentrations of C-reactive protein among patients with aortic stenosis [32]. However, the exact role of IL-6R polymorphisms is not clearly elucidated yet in inflammatory conditions, and further studies are warranted about this topic. There are some limitations that should be mentioned. First of all, the number of study participants was not high enough to make a generalization for such a common disease. Secondly, we only determined the genetic profile of cases with AA. IL-6 plays a crucial role in the pathophysiological process of inflammatory diseases, including AA. However, in our study IL-6 gene -572G/C and IL-6R gene 1:G.154448302 T > C polymorphisms did not play a critical role in pediatric cases with AA. However, multifactorial nature of AA may be misleading us to this result. Larger prospective studies are warranted to define the exact effects of these mutations in cytokine levels and inflammatory diseases.

## Conclusions

Given the putative biological importance of cytokine pathways, these data provide a new foundation to stimulate future debate and genetic investigations on AA which will focus on new diagnostic molecular mechanisms involving cytokine and cytokine receptor pathways. Additionally, these polymorphisms can be determined more easily from blood than inflammatory appendiceal tissue. Importantly, the present work underscores the need for further studies on identification and expression of functional SNPs in association with cytokine response, susceptibility to AA and prognostic factors. To the best of our knowledge, functional SNP variations on IL-6 and IL-6R in pediatric appendicitis patients in connection with AA have been reported here for the first time in the literature. We believe that outcomes of this study will reinforce interest and research in this hitherto neglected facet of AA research.

## Abbreviations

AA: Acute Appendicitis
CRP: C - reactive protein
ESR: erythrocyte sedimentation rate
HWE: Hardy-Weinberg equilibrium
SNP: single- nucleotide polymorphism
WBC: white blood cell
